# Do the Four Components of Psychological Capital Have Differential Buffering Effects? A Longitudinal Study on Parental Neglect and Adolescent Problematic Short-Form Video Use

**DOI:** 10.3390/bs15101396

**Published:** 2025-10-15

**Authors:** Lianpeng An, Xiaopan Xu, Hongwei Li, Qingqi Liu

**Affiliations:** 1 School of Law and Sociology, Luoyang Normal University, Luoyang 471934, China; musicann@163.com; 2 Institute for Public Policy and Social Management Innovation, College of Political Science and Public Administration, Henan Normal University, Xinxiang 453007, China; 3 School of History and Culture, Henan Normal University, Xinxiang 453007, China; 2013042@htu.edu.cn; 4 Department of Psychology, Faculty of Arts and Sciences, Beijing Normal University at Zhuhai, Zhuhai 519087, China; liuqingqi@bnu.edu.cn

**Keywords:** parental neglect, problematic short-form video use, psychological capital, self-efficacy, resilience, hope, optimism, longitudinal study

## Abstract

The growing prevalence of short-form video applications among adolescents has drawn increased public and scholarly attention to problematic short-form video use. The current longitudinal study gathered data from adolescents aged 12 to 15 across two waves spaced one year apart. A total of 665 participants provided reports on parental neglect, problematic short-form video use, psychological capital, and demographic details at Time 1 (T1), and reported again on problematic use at Time 2 (T2). After controlling for gender, age, parental education level, parental work status, family socioeconomic status, only-child status, and T1 problematic short-form video use, T1 parental neglect remained a significant predictor of T2 problematic use. Additionally, T1 self-efficacy, T1 resilience, and T1 hope significantly moderated the relationship between T1 parental neglect and T2 problematic use, whereas T1 optimism did not demonstrate a buffering effect. Specifically, the association between T1 parental neglect and T2 problematic use did not vary significantly between adolescents with high and low levels of optimism. However, the predictive effect was significantly weaker, though still statistically significant, among adolescents with higher self-efficacy and hope. Most notably, among those with higher resilience, the effect of parental neglect became non-significant. The study offers valuable evidence-based insights for preventing and addressing adolescent problematic short-form video use in the mobile internet era.

## 1. Introduction

In the mobile internet era, short-form video platforms have become an integral part of daily life for people worldwide. Although viewing short-form videos can provide enjoyment and relaxation, the potential risks of problematic usage and its adverse consequences should not be overlooked. The distinctive features of short-form videos readily trigger and intensify addictive behaviors among users. First, the pervasive availability of mobile devices allows short videos to fill any idle moment, often leading to habitual and unconscious scrolling behaviors. Second, short-form platforms utilize powerful AI algorithms to accurately identify user preferences, enabling an infinite scroll feature that removes natural stopping points and creates an endless, immersive viewing experience (Kim & Kim, 2024). More critically, short-form videos often incorporate high-intensity sensory stimuli. Lasting as little as 15 s, they deliver strong visual and auditory impact along with emotionally charged narratives, offering intense pleasure within brief periods and significantly reducing cognitive effort (Xiao et al., 2025). Substantial research indicates that problematic short-form video use poses serious threats to users’ physical and mental health, such as sleep disturbance (Yu et al., 2024), attention deficit (Chen et al., 2023), academic procrastination (J. Xie et al., 2023), depression (Zhu et al., 2024), and suicidal ideation and self-injurious behaviors (Yu et al., 2024).

Adolescents are among the most vulnerable groups for developing problem behaviors (Blum et al., 2002). Given their developmental stage, characterized by ongoing physical and mental maturation, they are more susceptible to various risk factors, which may lead to problematic internet use behaviors (Karacic & Oreskovic, 2017; Kuss et al., 2013). Research indicates that using diagnostic criteria for problematic short-form video use, more than 30% of adolescents meet the threshold for being classified as problematic users (Chao et al., 2023). In light of these detrimental effects and the high prevalence rates of problematic short-form video use, identifying key risk and protective factors, and examining how they interact in relation to problematic short-form video use, can offer important insights for understanding and effectively intervening in these behaviors. The present study will employ an ecological systems theory framework to examine the risk effects of parental neglect on adolescents’ problematic short-form video use, along with the protective role of psychological capital. Furthermore, it will compare the strength of the moderating effects across the four components of psychological capital to determine whether significant differences exist among them.

### 1.1. Parental Neglect and Problematic Short-Form Video Use

Parental neglect represents a significant form of harmful parenting behavior that adversely affects the development of children and adolescents (Maughan & Moore, 2010). It occurs when parents, despite possessing the necessary resources and capacity, fail to meet their children’s fundamental needs in critical areas such as physical health, emotional well-being, cognitive growth, or safety, ultimately impeding the child’s healthy development (Allin et al., 2005; Massullo et al., 2023). In contrast to active abusive behaviors, parental neglect primarily manifests as omission or inadequate response (Massullo et al., 2023). Parental neglect is typically categorized into two dimensions: physical neglect and emotional neglect (Cohen et al., 2017). Physical neglect involves the failure to provide essential material needs, including food, clothing, housing, healthcare, and a safe living environment (Stoltenborgh et al., 2013). Emotional neglect, on the other hand, pertains to the lack of necessary emotional support, affection, encouragement, and responsiveness (Proctor & Dubowitz, 2014). Extensive research has demonstrated that parental neglect can have profoundly negative effects on the physical and psychological development of children and adolescents. For example, studies indicate that parental neglect significantly increases the risk of depression among adolescents (Christ et al., 2017; Glickman et al., 2021), with these effects potentially enduring into adulthood (Bifulco et al., 2006). Additionally, parental neglect has been identified as a direct risk factor for problematic internet use behaviors in adolescents (Jameel et al., 2018; Kwak et al., 2018), with conditions such as internet gaming disorder (Gan et al., 2023; Shen et al., 2023; X. Xie et al., 2021) and problematic social media use (Chidambaram et al., 2023; J. Y. Zhang et al., 2025) being notably impacted by neglectful parenting.

Parental neglect may represent a significant risk factor for adolescents’ problematic use of short videos in the mobile internet era. Neglected adolescents frequently experience a lack of emotional warmth, acceptance, and recognition within their families, which may lead to a substantial emotional void in their lives. Such experiences frequently lead to persistent feelings of loneliness (Ayhan & Beyazit, 2021; Sun et al., 2024), anxiety (Kealy et al., 2023), and depression (Christ et al., 2017), while also limiting opportunities to learn effective emotional regulation strategies typically acquired through parental guidance (Beyazit et al., 2024; Hatkevich et al., 2021). Consequently, short-form video platforms offer a highly appealing compensatory mechanism. These platforms deliver instant gratification in an easily accessible format (David & Roberts, 2024; Zhu et al., 2024). Through rapid and high-intensity sensory stimulation, they trigger dopamine release, supplying momentary pleasure and serving as a digital analgesic for emotional distress (Y. Liu et al., 2021; C. Zhang & Zhu, 2025). Short-form videos can also create a sense of virtual belonging, fulfilling needs for social connection that may be unmet in offline environments and to alleviate loneliness that result from experiences of social exclusion. (Sarman & Tuncay, 2023; Taylor & Chen, 2024). Moreover, parental neglect often coincides with inadequate supervision and behavioral guidance (Allin et al., 2005; Massullo et al., 2023). Without parental modeling, many neglected children fail to develop crucial self-control ability such as goal-setting, time management, delayed gratification, and impulse control (Johnson & Vazsonyi, 2024; Ma & Chen, 2022; Z. Zhang et al., 2021). The structural design of short-video platforms further challenges self-regulation, featuring endless content flow and personalized algorithmic recommendations that promote continuous engagement. For adolescents with pre-existing self-control difficulties, resisting these engineered temptations proves especially challenging, often leading to uncontrolled and problematic usage behaviors.

Empirical research has established a significant relationship between parental neglect and adolescents’ problematic short-form video use (Q. Q. Liu et al., 2022a; H. Wang & Lei, 2022). For example, Q. Q. Liu et al. (2022a) demonstrated that parental neglect directly predicts adolescents’ problematic short-form video use behaviors. Similarly, H. Wang and Lei (2022) confirmed that parental phubbing (the behavior of parents becoming engrossed in their phones and neglecting their children), a prevalent form of parental neglect in the mobile internet era, not only has a direct predictive effect on adolescent problematic short-form video use but also generates an indirect predictive effect through the mechanism of relative deprivation. Furthermore, F. Li et al. (2023) found that parental neglect not only directly predicts problematic short-form video use among adolescents but also exerts an indirect influence through the mediating role of alexithymia. However, previous studies have primarily relied on cross-sectional survey designs, which limits the ability to rigorously establish the predictive effects of parental neglect, particularly over time. Building on prior theoretical and empirical research, we propose the following:

**Hypothesis** **1.** *Parental neglect will have a significant longitudinal predictive effect on adolescent problematic short-form video use.*

### 1.2. Protective Role of Psychological Capital

While parental neglect may exert significant long-term effects on adolescents’ problematic use of short-form videos, its influence is not necessarily absolute. Ecological systems theory highlights the intricate interactions between environmental and individual factors that collectively shape an individual’s physical and psychological development (Bronfenbrenner, 1979). Certain individual characteristics can serve a protective role, significantly mitigating the risk associated with parental neglect as a detrimental environmental influence. Among these positive individual factors, psychological capital is particularly noteworthy. Psychological capital represents a positive psychological state manifested by individuals throughout their growth and development (Luthans et al., 2007). It is an integrative concept that encompasses four key positive psychological factors: self-efficacy, resilience, optimism, and hope (Luthans & Youssef-Morgan, 2017). Self-efficacy refers to an individual’s confidence in their ability to complete tasks or cope with challenges (Bandura, 1978). In contrast, resilience denotes the capacity to adapt positively, cope effectively, and even recover and grow when confronted with significant adversity, trauma, tragedy, threat, or other major life stressors (Masten & Reed, 2002). It is important to note that among the four components of psychological capital, optimism and hope share a particularly close relationship. Both represent future-oriented, trait-like cognitive patterns. Some researchers have argued that conclusions attributed to hope could largely be ascribed to optimism (Aspinwall & Leaf, 2002), while others have conceptualized optimism as a subcomponent of hope (Peterson & Seligman). However, most contemporary research treats them as distinct constructs. Regarding optimism, most scholars adopt the concept of dispositional optimism, initially proposed by Scheier and Carver (1985), defining it as a generalized positive expectation that good rather than bad things will happen in the future. For hope, most scholars adopt Snyder’s (1994) conceptualization, defining it not merely as a positive future expectation but as a goal-directed thinking process comprising two core elements: pathway thinking (the capacity to generate routes to goals) and agency thinking (the motivational capacity to use those pathways). Therefore, beyond its future orientation, the conceptual core of hope lies in its focus on goal-directed agency and pathway thinking.

Psychological capital underscores the notion that positive psychological resources can be cultivated through interventions, enhancing personal resources (Luthans et al., 2008). The four components of psychological capital are interconnected and mutually reinforcing, collectively contributing to favorable outcomes. Research indicates that these components not only mitigate behavioral problems but also enhance mental health in adolescents (Finch et al., 2020; Gujar & Ali, 2019). In the context of problematic internet use, psychological capital plays a key protective role. Specifically, its four components not only are negative predictors of problematic internet use (Bi & Jin, 2021) but also mitigate the impact of risk factors (e.g., childhood trauma, neuroticism, and perceived stress) on these behaviors (Q. Liu et al., 2024; W. Wang et al., 2021; Yan et al., 2024).

Parental neglect deprives adolescents of critical psychological resources essential for their development, prompting them to seek compensation within the realm of short-form videos, which can lead to problematic use behaviors. In contrast, psychological capital offers vital internal resources, establishing a “psychological firewall” that effectively mitigates the negative consequences of neglect. Each component of psychological capital may play a distinct role in various contexts. Adolescents with a strong sense of self-efficacy believe they can manage academic tasks and daily challenges even without parental support (Franco & Rodrigues, 2018). They are inclined to confront issues directly and actively seek solutions, rather than retreating into short videos as a means of escape. Optimistic adolescents maintain positive expectations for the future, holding the belief that current difficulties are temporary, and things will improve (Rincon Uribe et al., 2022). The optimistic outlook fosters patience and motivation to endure present hardships, thus diminishing impulses toward instant gratification and the lure of immediate pleasures. Hopeful adolescents set meaningful goals independently (Esteves et al., 2013), such as gaining admission to a specific university or mastering a new skill. These well-defined objectives provide direction and a sense of purpose, rendering the appeal of short videos less significant. When faced with obstacles on their path to these goals, such as academic challenges, they do not easily surrender or resort to distraction; rather, they engage in critical thinking and explore various alternative solutions, such as consulting teachers, collaborating with peers, or researching online tutorials. The pathway thinking associated with hope can keep them actively engaged in the real world. Furthermore, resilient adolescents are adept at recovering from negative emotions, such as sadness and anger resulting from neglect, more swiftly (Fergus & Zimmerman, 2005), thereby reducing their reliance on binge-watching short videos as a form of “emotional first aid.” Moreover, resilience encompasses not only recovery but also post-traumatic growth (Anderson, 2018). These adolescents can interpret parental neglect as a formative experience, ultimately fostering greater independence, empathy, and a deeper understanding of life’s complexities. Based on the aforementioned theories and evidence, it is reasonable to hypothesize that all four components of psychological capital moderate the relationship between parental neglect and adolescents’ problematic short-form video use.

**Hypothesis** **2.** *Self-efficacy will significantly moderate the effect of parental neglect on adolescent problematic short-form video use*, *such that the effect will be weaker for adolescents with high self-efficacy.*

**Hypothesis** **3.** *Hope will significantly moderate the effect of parental neglect on adolescent problematic short-form video use*, *such that the effect will be weaker for adolescents with high hope*.

**Hypothesis** **4.** *Optimism will significantly moderate the effect of parental neglect on adolescent problematic short-form video use*, *such that the effect will be weaker for adolescents with high hope*.

**Hypothesis** **5.** *Resilience will significantly moderate the effect of parental neglect on adolescent problematic short-form video use*, *such that the effect will be weaker for adolescents with high resilience*.

### 1.3. The Present Study

Based on the individual-environment interaction model emphasized by ecological systems theory (Bronfenbrenner, 1979), our study aims to employ a two-wave longitudinal research design to investigate the interaction between parental neglect and psychological capital in relation to problematic short-form video use among adolescents. Specifically, we seek to determine whether the longitudinal predictive effect of parental neglect on adolescent problematic short-form video use is moderated by psychological capital. Building on our five confirmatory hypotheses, we introduce two exploratory research questions to examine the specific roles of the four psychological capital components as moderators. The exploratory approach is motivated by two considerations. First, given the substantial conceptual differences among the components, their buffering effects may vary in strength. Second, prior research suggests that the protective effect of optimism may be context-dependent (Okuzono et al., 2022; Segerstrom, 2005). Its future-oriented, generalized positive expectations might be less effective in countering the immediate gratifications of short-form videos compared to the goal-directed agency and pathways of hope, or the proactive coping embodied by self-efficacy and resilience. Therefore, we pose the following questions:

**Research Question** **1.** *Do the four components of psychological capital differ in the strength of their moderating effects on the relationship between parental neglect and adolescent problematic short-form video use?*

**Research Question** **2.** *How does the moderating effect of optimism compare to those of self-efficacy*, *hope*, *and resilience*?

## 2. Materials and Methods

### 2.1. Participants

Our study received approval from the Institutional Review Board at the corresponding author’s institution. Written informed consent was obtained from all participants. A two-wave prospective longitudinal study was conducted at three public middle schools in Central China, utilizing a researcher-designed questionnaire across two waves of data collection spaced one year apart. We employed a convenience sampling approach, selecting one public middle school in each of three cities: Wuhan, Hefei, and Xiangyang. These cities represent a range of regional development levels. Within each school, we randomly selected five classes per grade (grades seven to nine), resulting in 766 potential participants. Participation was voluntary, and students were excluded if they could not provide both personal and guardian informed consent. Our sample, drawn from public schools in these three central Chinese cities, captures key characteristics of the regional urban adolescent population. Over 95% of participants reported regular access to smartphones or tablets, consistent with high device penetration rates among Chinese adolescents. Thus, the sample appears reasonably representative of adolescents in urban public schools within central China, though caution should be exercised when generalizing to rural populations or students in elite private systems. In April 2024 (Time 1, T1), 717 adolescents aged 12 to 15 years completed the initial survey, which assessed demographic information, parental neglect, psychological capital, and problematic short-form video use. One year later, in April 2025 (Time 2, T2), 665 participants completed the follow-up survey, which again evaluated demographic characteristics and problematic short-form video usage. Data from these 665 participants who completed both waves were included in the final analysis. At the time of the first survey, participants’ ages ranged from 12 to 15 years (M = 13.19, SD = 1.04); the sample consisted of 349 boys and 316 girls. There were 266 seventh graders, 218 eighth graders, and 181 ninth graders. The specific demographic characteristics of the participants are presented in Table 1.

### 2.2. Measurements

#### 2.2.1. Parental Neglect

At Time 1, parental neglect was assessed with four items (e.g., “My parents left me alone, even though I needed to be with them”) adapted from the revised parental neglect subscale (Kwak et al., 2018) of the Parent–Child Conflict Tactics Scales (Straus et al., 1998). The Chinese version of the scale specifically measures emotional, physical, medical, and educational neglect. Participants reported how frequently they had experienced each form of neglect over the past year using a 5-point frequency scale (0 = never, 1 = 1–2 times, 2 = 3–5 times, 3 = 6–9 times, 4 = 10 or more times). Higher total scores reflect more severe levels of perceived parental neglect. In our study, the parental neglect scale demonstrated acceptable internal consistency, with a Cronbach’s α of 0.73.

#### 2.2.2. Problematic Short-Form Video Use

To ensure conceptual clarity, we adopt the term problematic short-form video use. The terminology “problematic use” reflects a behavioral perspective that emphasizes maladaptive patterns of use and their functional impairments, distinguishing it from a pathologizing framework of clinical addiction. Problematic short-form video use was assessed at both time points with seven items (e.g., “I have no concept of time at all when I view short-form videos on my phone”) from the Mobile Short-Form Video Addiction subscale of the Mobile Phone Addiction Type Scale (MPATS) (Q. Q. Liu et al., 2022b). Participants rated each item on a five-point scale ranging from 1 (never) to 5 (always). The scale measures core dimensions of problematic mobile phone use, including loss of control, negative emotions due to restricted use, withdrawal/escape tendencies, and functional impairment. Higher total scores reflect more severe problematic short-form video use. The scale demonstrated high internal consistency, with Cronbach’s alpha values of 0.88 at Time 1 and 0.94 at Time 2.

#### 2.2.3. Psychological Capital

At Time 1, psychological capital was measured using the Chinese version of the Positive Psychological Capital Questionnaire (K. Zhang et al., 2010), which was adapted from Luthans et al.’s (2007) Psychological Capital Questionnaire (PCQ). The scale consists of 26 items rated on a 7-point Likert-type scale, ranging from 1 (strongly disagree) to 7 (strongly agree), assessing four dimensions of psychological capital. Specifically, seven items measure self-efficacy, seven measure resilience, six measure hope, and six measure optimism. Higher scores on each subscale indicate stronger levels of the corresponding psychological capital component, and higher total scores reflect greater overall psychological capital. The Chinese Positive Psychological Capital Questionnaire we employed is a culturally adapted version developed by K. Zhang et al. (2010), building upon but substantially modifying the original PCQ by Luthans et al. (2007). The adaptation was undertaken to address two primary limitations of the original: its initial design for a workforce population and its suboptimal test–retest reliability due to a strong focus on transient states. To enhance its cultural relevance and psychometric robustness for the Chinese population, K. Zhang et al. (2010) developed new items and integrated them with original PCQ items. The final 26-item scale was validated through exploratory and confirmatory factor analyses, demonstrating strong reliability and validity. Compared to Luthans et al.’s (2007) PCQ, the Chinese version removed phrases that overly emphasized professional contexts, making it more applicable to the general Chinese population. The scale has demonstrated good reliability and validity in Chinese adolescent and adult populations (Y. Li & Zhu, 2024; R. Song & Song, 2021; W. Wang et al., 2021). In our study, the subscales demonstrated good internal consistency, with Cronbach’s alpha values of 0.93 for self-efficacy, 0.93 for resilience, 0.88 for hope, and 0.87 for optimism.

### 2.3. Statistical Analysis

We first employed descriptive statistics to characterize the core variables. We then performed Pearson correlation analysis to evaluate the bivariate relationships among the key variables, paying particular attention to the longitudinal association between parental neglect and adolescents’ problematic short-form video use. Finally, we used Hayes’s (2013) PROCESS macro for SPSS (version 23.0) to examine the moderating effects of four psychological capital components (i.e., self-efficacy, resilience, hope, and optimism) on the longitudinal relationship between parental neglect and adolescent problematic short-form video use. The variance inflation factor (VIF) values for the PsyCap components were as follows: self-efficacy (1.78), hope (1.70), optimism (1.71), and resilience (1.72). All values are well within acceptable limits (VIF < 5), confirming that multicollinearity does not unduly influence our results. Furthermore, consistent with best practices for moderation analysis, the predictor variables involved in the interaction terms were mean-centered to reduce non-essential multicollinearity and improve the interpretability of the interactions. Gender, age, parental education level, parental work status, family socioeconomic status, and only-child status were included as covariates in all models to control for their potential confounding effects.

## 3. Results

### 3.1. Preliminary Analysis

Table 2 displays the correlations among the core variables. Parental neglect was positively correlated with problematic short-form video use at both Time 1 (*r* = 0.46, *p* < 0.001) and Time 2 (*r* = 0.50, *p* < 0.001). However, it showed significant negative correlations with the four psychological capital factors: self-efficacy (*r* = −0.25, *p* < 0.001), resilience (*r* = −0.34, *p* < 0.001), hope (*r* = −0.24, *p* < 0.001), and optimism (*r* = −0.17, *p* < 0.001). In turn, all of these factors were also negatively correlated with T2 problematic short-form video use (self-efficacy: *r* = −0.36; resilience: *r* = −0.37; hope: *r* = −0.26; optimism: *r* = −0.24; all *p* values < 0.001).

### 3.2. Moderating Effect Analysis

**Figure 1:**
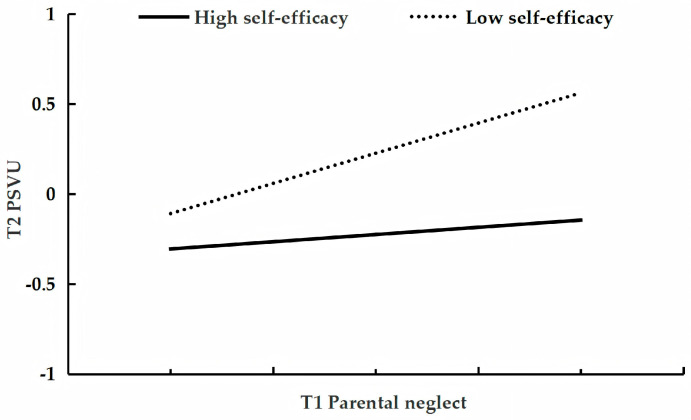
The relationship between T1 parental neglect and T2 problematic short-form video use at low and high levels of self-efficacy.

Table 3 presents the results of the analysis testing the moderating role of self-efficacy in the longitudinal relationship between parental neglect and adolescent problematic short-form video use. After controlling for gender, age, parental education level, parental work status, family socioeconomic status, and only-child status, and T1 problematic short-form video use, T1 parental neglect was a significant positive predictor of T2 problematic use (β = 0.23, *p* < 0.001, 95%CI [0.15, 0.30]). In contrast, T1 self-efficacy was a significant negative predictor (β = −0.16, *p* < 0.001, 95%CI [−0.23, −0.09]). Critically, the interaction between T1 parental neglect and T1 self-efficacy was also a significant negative predictor (β = −0.10, *p* < 0.001, 95%CI [−0.16, −0.05]). Conditional effect analysis revealed that although parental neglect significantly predicted problematic use at both high and low levels of self-efficacy, its effect was stronger among adolescents with low self-efficacy (β = 0.33, *p* < 0.001, 95% CI [0.24, 0.42]) than among those with high self-efficacy (β = 0.12, *p* < 0.05, 95% CI [0.02, 0.22]; see Figure 1).

**Figure 2:**
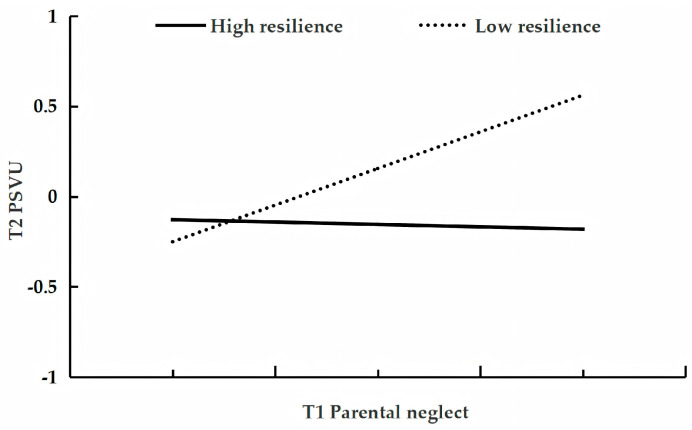
The relationship between T1 parental neglect and T2 problematic short-form video use at low and high levels of resilience.

Table 4 presents the results testing the moderating role of resilience in the longitudinal relationship between parental neglect and adolescent problematic short-form video use. After controlling for gender, age, parental education level, parental work status, family socioeconomic status, and only-child status, and T1 problematic short-form video use, the analysis revealed that T1 parental neglect was a positive predictor of T2 problematic use (β = 0.21, *p* < 0.001, 95%CI [0.14, 0.28]), while T1 resilience was a negative predictor (β = −0.11, *p* < 0.01, 95%CI [−0.18, −0.05]). Furthermore, the interaction between T1 parental neglect and T1 resilience was a significant negative predictor of T2 problematic use (β = −0.17, *p* < 0.001, 95%CI [−0.22, −0.11]). Conditional effect analysis showed that the effect of T1 parental neglect on T2 use was significant among adolescents with low resilience (β = 0.37, *p* < 0.001, 95%CI [0.29, 0.45]) but became non-significant among those with high resilience (β = 0.04, *p* = 0.78, 95%CI [−0.06, 0.14]; see Figure 2).

**Figure 3:**
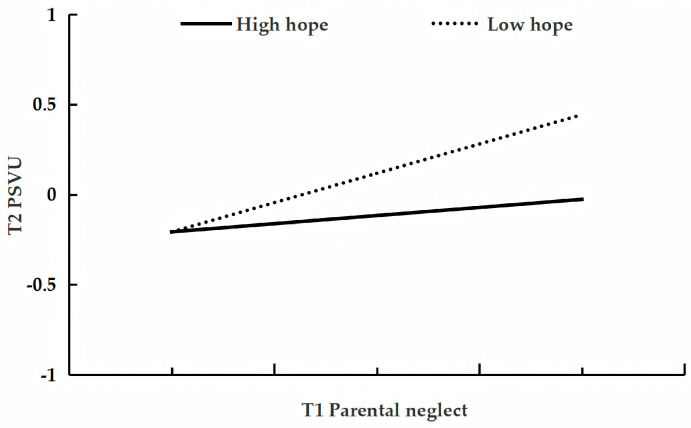
The relationship between T1 parental neglect and T2 problematic short-form video use at low and high levels of hope.

Table 5 presents the analysis testing hope as a moderator of the longitudinal relationship between parental neglect and adolescent problematic short-form video use. After controlling for gender, age, parental education level, parental work status, family socioeconomic status, and only-child status, and T1 problematic short-form video use, T1 parental neglect positively predicted T2 use (β = 0.23, *p* < 0.001, 95%CI [0.15, 0.30]), whereas T1 hope was a negative predictor (β = −0.09, *p* < 0.01, 95%CI [−0.15, −0.02]). The interaction between T1 parental neglect and T1 hope was also a significant negative predictor (β = −0.10, *p* < 0.001, 95%CI [−0.16, −0.05]). Conditional effect analysis revealed that although parental neglect significantly predicted T2 use at both levels of hope, its effect was stronger in the low-hope group (β = 0.33, *p* < 0.001, 95%CI [0.25, 0.41]) than in the high-hope group (β = 0.12, *p* < 0.05, 95%CI [0.02, 0.22]; see Figure 3).

**Figure 4:**
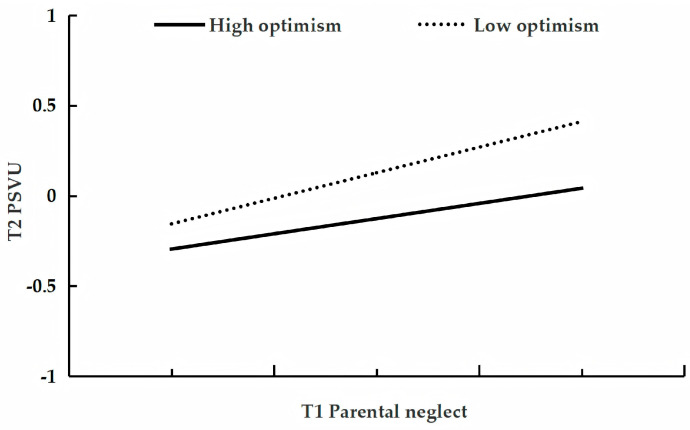
The relationship between T1 parental neglect and T2 problematic short-form video use at low and high levels of optimism.

Table 6 presents the results of the analysis testing the moderating role of optimism in the longitudinal relationship between parental neglect and adolescent problematic short-form video use. After controlling for gender, age, parental education level, parental work status, family socioeconomic status, and only-child status, and T1 problematic use, the analysis revealed that T1 parental neglect was a positive predictor of T2 use (β = 0.25, *p* < 0.001, 95%CI [0.18, 0.33]), while T1 optimism was a negative predictor (β = −0.10, *p* < 0.01, 95%CI [−0.17, −0.03]). However, the interaction between T1 parental neglect and T1 optimism was not a significant predictor of T2 problematic use (β = −0.05, *p* = 0.09, 95%CI [−0.12, 0.01]). Consequently, simple slope analysis showed that the effect of T1 parental neglect on T2 use did not differ significantly between adolescents with low (β = 0.31, *p* < 0.001, 95%CI [0.20, 0.41]) and high levels of optimism (β = 0.20, *p* < 0.001, 95%CI [0.10, 0.29]; see Figure 4).

## 4. Discussion

The widespread adoption of various short-form video applications has positioned adolescents as a key population vulnerable to developing problematic usage patterns. Our study investigated the longitudinal effect of parental neglect on adolescent problematic short-form video use. It examined whether all four components of positive psychological capital serve as significant buffers and compared their moderating roles. Results indicated that parental neglect significantly predicted problematic short-form video use one year later. Moreover, psychological capital, particularly three of its components, played a critical buffering role by alleviating the effect of parental neglect. Specifically, self-efficacy, resilience, and hope each significantly attenuated the predictive effect of parental neglect on problematic use, whereas optimism did not demonstrate a significant buffering effect. The association between parental neglect and problematic use did not differ significantly between adolescents with high versus low levels of optimism. However, the effect was weaker, though still significant, among those with higher self-efficacy and hope. Most notably, among adolescents with higher resilience, the effect of parental neglect became statistically non-significant. These findings not only confirm the long-term effect of parental neglect on adolescent problematic short-form video use but also clarify the distinct moderating roles played by different components of psychological capital. The study offers valuable practical insights for preventing and intervening in problematic short-form video use among adolescents, especially those susceptible to parental neglect.

Consistent with Hypothesis 1, we found that parental neglect remained a significant predictor of problematic short-form video use one year later, even after controlling for gender, age, and baseline levels of such use. The result aligns with previous research on parental neglect and adolescent problematic internet use (Jameel et al., 2018; Kwak et al., 2018), particularly studies focusing on short-form video use (F. Li et al., 2023; Q. Q. Liu et al., 2022a; H. Wang & Lei, 2022). The current study extends prior work through its one-year longitudinal design, which supports stronger causal inferences regarding the relationship between parental neglect and problematic short-form video use, especially its long-term predictive effect. Our results largely support applying the psychological decompensation theory (Wu et al., 2019) to adolescent problematic short-form video use. As posited by the psychological decompensation theory (Wu et al., 2019), when core psychological needs, including autonomy, competence, and relatedness, are unmet in real life, individuals may turn to excessive internet use to compensate for these deficits, potentially leading to addictive behaviors. Parents represent one of the most important sources for fulfilling adolescents’ basic psychological needs. Parental neglect directly undermines and obstructs these developmental requirements (J. Y. Zhang et al., 2025). Such neglect often leads children to feel unnoticed, unloved, and emotionally disconnected, thereby damaging their sense of relatedness (Ho & Schermer, 2024). It also results in lack of recognition for achievements and inadequate support during challenges, which diminishes self-worth and frustrates competence needs (Soffer et al., 2008). Furthermore, neglect typically involves absence of positive guidance and role models, hindering the development of healthy self-regulation and emotion management strategies (Beyazit et al., 2024). That pattern heightens dependence on automatic and impulsive emotional and behavioral responses, further eroding autonomy. Short-form video platforms, however, readily provide an alternative means of satisfying these psychological needs. Initially, short-form video use may represent a conscious compensatory strategy. However, because it is highly accessible and immediately rewarding, adolescents may increasingly rely on it while disengaging from constructive real-world problem-solving. Over time, such compensatory use can develop into problematic short-form video use.

Consistent with Hypotheses 2, 3, and 4, the present study confirmed that three components of positive psychological capital (i.e., self-efficacy, resilience, and hope) served as significant protective factors that buffered the long-term effect of parental neglect on adolescent problematic short-form video use. However, the buffering effect of optimism was not significant; thus, Hypothesis 5 was not supported. Although previous research has examined the moderating role of psychological capital in the relationship between negative parenting and its negative outcomes (C. Song et al., 2025), no study to date has directly tested whether psychological capital moderates the influence of parental neglect on adolescent problematic short-form video use, nor compared the potential differential roles of its individual components. Our findings demonstrate that the moderating effects of the components of psychological capital in the relationship between parental neglect and problematic short-form video use are distinct. The buffering role of psychological capital is not a uniform “all-or-nothing” effect, but rather varies across its constituent dimensions.

In our study, self-efficacy and hope demonstrated similar patterns of moderating effects. Compared to adolescents with lower levels of self-efficacy or hope, the predictive effect of parental neglect on problematic short-form video use was weaker among those with higher levels of either trait, although the association remained statistically significant. One core harm of parental neglect lies in its erosion of external support resources through emotional absence and lack of guidance (Maughan & Moore, 2010), which may lead adolescents to compensate by relying on the immediate comfort of short-form videos, as they lack sufficient external resources to cope with negative emotions like loneliness and anxiety. Adolescents with high self-efficacy believe in their ability to manage loneliness, regulate emotions, and resist the temptation of short-form videos. They are therefore more likely to employ active coping strategies—such as physical exercise, talking with others, or journaling—to alleviate negative emotions (Schunk et al., 2022) and effectively regulate their short-form video use behaviors. Similarly, adolescents with high hope can maintain commitment to their goals and develop pathways to achieve them even in the context of parental neglect (Kwok & Gu, 2019; Sparks et al., 2021). Their positive pursuit of future objectives channels time and energy toward meaningful pursuits rather than excessive short-form video consumption. In summary, both self-efficacy and hope compensate for the lack of external support caused by parental neglect by enhancing internal resources, thereby attenuating its predictive effect on problematic use. Nevertheless, the negative influence of parental neglect remained significant even among adolescents with higher levels of self-efficacy or hope, indicating that these traits function by “weakening” rather than “eliminating” its detrimental effects. It is possible that their protective power could be enhanced when combined with other psychological or environmental resources.

In contrast to the moderating effects of self-efficacy and hope, resilience demonstrated a stronger protective role. The long-term predictive effect of parental neglect on adolescent problematic short-form video use was significant only among adolescents with low resilience and became non-significant among those with high levels of resilience. Unlike self-efficacy, which reflects belief in one’s capabilities, or hope, which involves goal-directed thinking and pathway planning, resilience directly manifests in actual recovery behaviors (Luthans et al., 2007). The core of resilience lies in the capacity for both immunity to and recovery from adverse events (Herrman et al., 2011). When confronted with parental neglect, adolescents with high resilience can quickly restore psychological equilibrium through adaptive strategies such as engaging in hobbies or interests, even after experiencing short-term emotional distress. Resilience thus helps reduce their reliance on the immediate stimulation offered by short-form videos. Some individuals may even reframe parental neglect as a form of tempering that can be transformed into motivation for growth, thereby fostering more autonomous, independent, and positive personality traits. It is the ability to recover from adversity, setbacks, and failures, and potentially emerge stronger, that allows highly resilient adolescents to minimize the negative impact of parental neglect.

Interestingly, our findings indicate that optimism did not serve a significant moderating role in the relationship between parental neglect and adolescent problematic short-form video use. Parental neglect often elicits immediate and sustained emotional distress (Christ et al., 2017; Kealy et al., 2023), and short-form videos provide a highly accessible source of pleasure that alleviates such pain in the present moment. However, optimism entails a positive expectation for the future (Bruininks & Malle, 2005) but does not, by itself, drive the goal-setting and proactive effort characteristic of hope. Consequently, the forward-looking perspective alone fails to mitigate the immediate sadness caused by emotional neglect, and the resulting negative emotions may still drive adolescents to seek short-term relief through short-form videos, leading to excessive use. In contrast, hope encompasses not only future-oriented positivity but also goal-directed thinking—specifically, agency thinking (the motivation to pursue goals) and pathway thinking (the capacity to generate strategies to achieve them) (Snyder, 2002). These components empower individuals to actively select effective means and take constructive action. The significant regulatory role of hope, compared to the non-significant role of optimism, suggests that merely holding positive future expectations is insufficient for addressing serious real-world issues; effective action driven by clear goals is essential to buffer the impact of negative events. Moreover, although the Chinese version of the Psychological Capital Questionnaire we employed attempts to address the original PCQ’s stronger emphasis on state-like rather than trait-like characteristics, the use of self-report measures may still incompletely capture an individual’s stable positive psychological traits. If the optimism measured in our study reflects more of a transient state than a stable disposition, its moderating effect could be attenuated or non-significant. Furthermore, although optimism is generally adaptive, it may function as a dual-factor influence in contexts of persistent adversity that demand active resolution. This is particularly evident when optimism becomes excessive or unrealistic. For instance, if adolescents attribute parental neglect to externally justified reasons (e.g., believing their parents are too busy with work), they may avoid initiating communication or expressing emotional needs. Such avoidance can unintentionally sustain the neglectful dynamic and foster reliance on short-form videos as a form of avoidant coping. Additionally, they may optimistically overestimate that frequent short-form video use will not harm their academic or daily functioning, thereby increasing the risk of problematic use. Thus, in situations of profound emotional neglect, optimism may fail to alleviate immediate distress and could even promote dependence on short-form videos for instant relief.

The present study has several limitations. First, the research focused exclusively on adolescents aged 12 to 15 and did not include children, who are also at high risk for problematic short-form video use. Future studies could examine whether the four components of positive psychological capital buffer the relationship between parental neglect and problematic short-form video use in child samples, or conduct comparative analyses involving both children and adolescents. Second, although a one-year longitudinal design was employed, the correlational nature of the data precludes definitive causal inferences. Future research should utilize quasi-experimental intervention designs to test whether psychological capital interventions significantly reduce problematic short-form video use among neglected adolescents. Thirdly, the study only examined the buffering role of the four psychological capital components in the direct effect of parental neglect on problematic use, without examining potential longitudinal mediation pathways or their moderated mediation (i.e., conditional indirect) effects. Parental neglect may influence problematic short-form video use both directly and through indirect pathways (F. Li et al., 2023; H. Wang & Lei, 2022). Future studies should investigate how parental neglect is longitudinally associated with adolescent short-form video use and whether psychological capital moderates both the direct and indirect effects in this longitudinal process. Finally, while our study focused specifically on the role of parental neglect, it is important to acknowledge that adolescent behavior is shaped by a multitude of factors. Variables such as peer influence, academic performance, and broader familial or environmental stressors were beyond the scope of the present investigation but represent critical avenues for future research. Examining these factors could further elucidate the complex mechanisms underlying the relationship between parental neglect and problematic media use.

Despite the aforementioned limitations, our study offers valuable practical implications for the prevention and intervention of adolescent problematic short-form video use. First, parental neglect is not only closely associated with such problematic use but also exerts a stable long-term influence. It is therefore important to raise parental awareness regarding the negative impact of neglectful behaviors. Public media campaigns could help emphasize the detrimental effects of parental neglect on child development. Government agencies may consider implementing parent education programs or workshops to teach caregivers how to provide emotional responsiveness, positive attention, and appropriate supervision, thereby reducing parental neglect at its source. Second, schools and teachers should integrate psychological capital education into curricula and daily activities. To cultivate self-efficacy, educators can facilitate micro-success experiences, skill-building tasks, and role-model learning, all of which help students develop confidence through accumulated mastery. Specific strategies include goal decomposition—breaking long-term objectives into manageable steps—and self-monitoring training, such as using digital tools to track and reflect on progress in time management, thereby systematically building a sense of competence. To foster hope, students should be guided to set personalized and meaningful goals while practicing pathways thinking: identifying multiple routes to a desired outcome (e.g., improving a grade) and anticipating potential obstacles. These strategies can strengthen both the capacity to envision alternatives and the motivation to pursue them. To enhance resilience, it is essential to incorporate problem-solving training that helps adolescents identify stressors—such as those related to parental neglect or school life—generate alternative solutions, and evaluate outcomes. Additionally, teaching emotion regulation skills (e.g., mindfulness, emotional labeling) and cognitive reappraisal techniques to reframe setbacks positively, along with encouraging help-seeking behaviors, can significantly bolster resilience (Jahantigh et al., 2024; J. Y. Zhang et al., 2022). Given that resilience demonstrated a stronger protective effect than self-efficacy and hope in our study, intervention programs should place particular emphasis on activities that promote learning and growth from adversity. Furthermore, efforts to cultivate psychological capital should avoid promoting blind or passive optimism. Instead, they should foster a reality-based, action-oriented form of optimism that helps adolescents recognize that although difficulties exist, proactive effort and problem-solving can lead to tangible improvements. Integrating optimism with self-efficacy and hope may thus yield more powerful outcomes. In summary, our findings suggest that merely restricting short-form video use may address symptoms rather than root causes. A more effective approach may involve compensating for the emotional void and unmet developmental needs resulting from parental neglect through healthy, authentic resources. Prevention and intervention efforts should therefore shift emphasis from risk reduction, while still necessary, to the systematic enhancement of protective factors, specifically through the cultivation of adolescents’ positive psychological traits.

## 5. Conclusions

The present study employed a longitudinal questionnaire design to examine the predictive effect of parental neglect on adolescents’ problematic short-form video use over time and to investigate the potential buffering role of the four components of psychological capital. The findings revealed that parental neglect significantly predicted levels of problematic short-form video use one year later. Self-efficacy, resilience, and hope each significantly buffered the long-term predictive effect of parental neglect on problematic use, whereas optimism did not demonstrate a significant moderating effect. Specifically, the association between parental neglect and problematic short-form video use did not differ significantly across adolescents with high versus low levels of optimism. However, the longitudinal effect of parental neglect was significantly weaker (though still significant) among adolescents with higher self-efficacy and hope. Notably, among adolescents with higher resilience, the predictive effect of parental neglect on problematic short-form video use became non-significant. These results extend previous research on the detrimental effects of parental neglect by highlighting the protective role of psychological capital and underscoring the differential moderating effects of its specific components. The findings can offer scientifically grounded insights for the prevention and intervention of adolescent problematic short-form video use.

## Figures and Tables

**Table 1:** Demographic information of the participants.

Characteristics	Categories	Number (Percentage)
Gender	Male	349 (52.5%)
	Female	316 (47.5%)
Age	12 years old	221 (33.2%)
	13 years old	188 (28.3%)
	14 years old	166 (25.0%)
	15 years old	90 (13.5%)
Grade	Seventh grade	266 (40.0%)
	Eighth grade	218 (32.8%)
	Ninth grade	181 (27.2%)
Maternal education level	Junior high school or below	190 (28.6%)
	Senior high school or vocational school	172 (25.9%)
	College (associate or bachelor’s degree)	187 (28.1%)
	Master’s degree or above	116 (17.4%)
Paternal education level	Junior high school or below	164 (24.7%)
	Senior high school or vocational school	170 (25.6%)
	College (associate or bachelor’s degree)	214 (32.2%)
	Master’s degree or above	117 (17.6%)
Maternal work status	Unemployed	72 (10.8%)
	Part-time employed	71 (10.7%)
	Full-time employed	522 (78.5%)
Paternal work status	Unemployed	51 (7.7%)
	Part-time employed	94 (14.1%)
	Full-time employed	520 (78.2%)
Family socioeconomic status	Very low	131 (20.3%)
	Relatively low	181 (27.2%)
	Medium	215 (32.3%)
	Relatively high	69 (10.4%)
	Very high	65 (9.8%)
Only child	Yes	410 (61.7%)
	No	255 (38.3%)

**Table 2:** Descriptive statistics and correlations between variables.

Variables	1	2	3	4	5	6	7
1. T1 Parental neglect	—						
2. T1 Self-efficacy	−0.25 ***	—					
3. T1 Resilience	−0.34 ***	0.55 ***	—				
4. T1 Hope	−0.24 ***	0.54 ***	0.55 ***	—			
5. T1 Optimism	−0.17 ***	0.56 ***	0.53 ***	0.52 ***	—		
6. T1 PSVU	0.46 ***	−0.29 ***	−0.34 ***	−0.20 ***	−0.18 ***	—	
7. T2 PSVU	0.50 ***	−0.36 ***	−0.37 ***	−0.26 ***	−0.24 ***	0.60 ***	—
Mean	5.02	32.02	34.10	29.37	28.17	13.13	14.64
SD	3.61	9.88	9.86	7.81	7.63	6.76	7.84

Note. *N* = 665. PSVU = Problematic short-form video use. *** *p* < 0.001.

**Table 3:** Moderating effect of self-efficacy in the relationship between T1 parental neglect and T2 problematic short-form video use.

Regression Equation		Significance of Coefficients				Bootstrap	
Outcome Variable	Independent Variables	β	SE	*t*	*p*	LLCI	ULCI
T2 PSVU	Gender	−0.06	0.06	−1.02	0.31	−0.17	0.05
	Age	−0.08 **	0.03	−2.75	<0.01	−0.14	−0.02
	Maternal education level	−0.21	0.03	6.20	<0.001	−0.27	−0.14
	Paternal education level	0.14	0.03	4.33	<0.001	0.08	0.21
	Maternal work status	0.03	0.03	0.88	0.38	−0.03	0.09
	Paternal work status	0.01	0.04	0.39	0.70	−0.06	0.08
	Family socioeconomic status	0.05	0.03	1.92	0.06	−0.01	0.10
	Only child	−0.02	0.06	−0.28	0.78	−0.14	0.11
	T1 PSVU	0.39 ***	0.04	9.97	<0.001	0.31	0.46
	T1 Parental neglect	0.23 ***	0.04	6.08	<0.001	0.15	0.30
	T1 Self-efficacy	−0.16 ***	0.03	−4.59	<0.001	−0.23	−0.09
	T1 Parental neglect × T1 Self-efficacy	−0.10 ***	0.03	−3.47	<0.001	−0.16	−0.05

Note. *N* = 665. PSVU = Problematic short-form video use. Bootstrap sample size = 5000. LL = low limit, CI = confidence interval, UL = upper limit. ** *p* < 0.01. *** *p* < 0.001.

**Table 4:** Moderating effect of resilience in the relationship between T1 parental neglect and T2 problematic short-form video use.

Regression Equation		Significance of Coefficients				Bootstrap	
Outcome Variable	Independent Variables	β	SE	*t*	*p*	LLCI	ULCI
T2 PSVU	Gender	−0.08	0.06	−1.40	0.16	−0.19	0.03
	Age	−0.08 **	0.03	−2.86	<0.01	−0.14	−0.03
	Maternal education level	−0.21 ***	0.03	−6.28	<0.001	−0.27	−0.14
	Paternal education level	0.15 ***	0.03	4.76	<0.001	0.09	0.22
	Maternal work status	0.02	0.03	0.73	0.47	−0.04	0.08
	Paternal work status	0.04	0.03	1.05	0.29	−0.03	0.10
	Family socioeconomic status	0.05 *	0.03	2.16	<0.05	0.01	0.10
	Only child	−0.02	0.06	−0.35	0.73	−0.15	0.10
	T1 PSVU	0.36 ***	0.04	9.20	<0.001	0.28	0.43
	T1 Parental neglect	0.21 ***	0.04	5.77	<0.001	0.14	0.28
	T1 Resilience	−0.11 **	0.03	−3.32	<0.01	−0.18	−0.05
	T1 Parental neglect × T1 Resilience	−0.17 ***	0.03	−5.97	<0.001	−0.22	−0.11

Note. *N* = 665. PSVU = Problematic short-form video use. Bootstrap sample size = 5000. LL = low limit, CI = confidence interval, UL = upper limit. * *p* < 0.05. ** *p* < 0.01. *** *p* < 0.001.

**Table 5:** Moderating effect of hope in the relationship between T1 parental neglect and T2 problematic short-form video use.

Regression Equation		Significance of Coefficients				Bootstrap	
Outcome Variable	Independent Variables	β	SE	*t*	*p*	LLCI	ULCI
T2 PSVU	Gender	−0.07	0.06	−1.21	0.23	−0.18	0.04
	Age	−0.07 *	0.03	−2.47	<0.05	−0.13	−0.02
	Maternal education level	−0.22 ***	0.03	−6.55	<0.001	−0.28	−0.15
	Paternal education level	0.16 ***	0.03	4.74	<0.001	0.09	0.22
	Maternal work status	0.02	0.03	0.82	0.41	−0.03	0.08
	Paternal work status	0.02	0.04	0.57	0.57	−0.05	0.09
	Family socioeconomic status	0.05	0.03	1.82	0.07	−0.01	0.10
	Only child	−0.02	0.06	−0.29	0.77	−0.15	0.11
	T1 PSVU	0.42 ***	0.04	11.21	<0.001	0.35	0.49
	T1 Parental neglect	0.23 ***	0.04	6.03	<0.001	0.15	0.30
	T1 Hope	−0.09 **	0.03	−2.74	<0.01	−0.15	−0.02
	T1 Parental neglect × T1 Hope	−0.10 ***	0.03	−3.94	<0.001	−0.16	−0.05

Note. *N* = 665. PSVU = Problematic short-form video use. Bootstrap sample size = 5000. LL = low limit, CI = confidence interval, UL = upper limit. * *p* < 0.05. ** *p* < 0.01. *** *p* < 0.001.

**Table 6:** Moderating effect of optimism in the relationship between T1 parental neglect and T2 problematic short-form video use.

Regression Equation		Significance of Coefficients				Bootstrap	
Outcome Variable	Independent Variables	β	SE	*t*	*p*	LLCI	ULCI
T2 PSVU	Gender	−0.06	0.06	−1.04	0.30	−0.17	0.05
	Age	−0.07 *	0.03	−2.50	<0.05	−0.13	−0.02
	Maternal education level	−0.21	0.03	−6.16	<0.001	−0.28	−0.14
	Paternal education level	0.15	0.03	4.37	<0.001	0.08	0.22
	Maternal work status	0.02	0.03	0.56	0.57	−0.04	0.08
	Paternal work status	0.02	0.04	0.56	0.57	−0.05	0.09
	Family socioeconomic status	0.04	0.03	1.38	0.17	−0.02	0.09
	Only child	−0.02	0.06	−0.34	0.74	−0.15	0.11
	T1 PSVU	0.42 ***	0.04	11.01	<0.001	0.35	0.50
	T1 Parental neglect	0.25 ***	0.04	6.56	<0.001	0.18	0.33
	T1 Optimism	−0.10 **	0.04	−2.76	<0.01	−0.17	−0.03
	T1 Parental neglect × T1 Optimism	−0.05	0.03	−1.68	0.09	−0.12	0.01

Note. *N* = 665. PSVU = Problematic short-form video use. Bootstrap sample size = 5000. LL = low limit, CI = confidence interval, UL = upper limit. * *p* < 0.05. ** *p* < 0.01. *** *p* < 0.001.

## Data Availability

The data are not publicly available due to privacy and ethical restrictions. The data that support the findings of this study are available on reasonable request from the corresponding author following the completion of a privacy and fair use agreement.
